# Why are there so few pathogens? Ecology and evolution in pathogen emergence

**DOI:** 10.1371/journal.pbio.3003329

**Published:** 2025-08-21

**Authors:** John M. Drake

**Affiliations:** 1 Odum School of Ecology and Center for the Ecology of Infectious Diseases, University of Georgia, Athens, Georgia, United States of America; 2 Pandemic Sciences Institute and Oxford Martin School, University of Oxford, Oxford, United Kingdom

## Abstract

Why are there so few pathogens, and what determines their emergence? This Perspective argues that ecological and evolutionary forces (host availability, geographic exposure and microbial innovation) will shape future human diseases.

## Teaser

Why are there so few pathogens, and what determines their emergence? The ecological and evolutionary forces of host availability, geographic exposure, and microbial innovation will shape future human diseases.

The fraction of microorganisms that are pathogenic to any given host species is minuscule, as shown in a recent comprehensive study that identified just 1,513 bacterial species that are pathogenic in humans, surely the most thoroughly studied of all vertebrate hosts [1]. In his recent Perspective article [2], Arturo Casadevall asked: what traits, then, explain why some microorganisms are pathogenic while most are not? He proposed a three-part answer. To be pathogenic to humans, a microorganism must simultaneously possess: the ability to survive within the host environment; virulence factors that allow it to infect the host and resist immune eradication; and host susceptibility, particularly immunological history, genetics and the presence of receptors for attachment and host cell invasion. In addition to these factors, the microorganism must damage the host, imposing a fitness cost. Because this confluence of traits is both rare and often transient, Casadevall concluded that “the capacity for virulence is such a demanding phenotype that it is rare and can be fleeting” [2].

These biological attributes of microorganism and host are only half of the story, however, as pathogen emergence is not solely a biological event, but also an ecological and evolutionary one. It may be that there are so few pathogens because there has not been sufficient opportunity. In particular, I propose expanding the framework presented by Casadevall to include three additional factors: host availability (i.e., the abundance of potential hosts and demographic and social structures that make hosts accessible to pathogens); exposure of hosts to pathogens, which requires microorganisms to occupy environmental and geographic distributions that cause them to encounter potential hosts; and opportunity for genomic innovation (i.e., evolutionary mechanisms such as horizontal gene transfer (HGT) that enable microorganisms to acquire and retain traits associated with cell entry, immune escape, drug resistance or pathology).

Instead of Casadevall’s Venn diagram with a tiny intersection, I propose a new framework (Table 1). On the left-hand side are the necessary biological conditions (the Casadevall model [2]). On the right-hand side are the ecological and evolutionary accelerants. The latter—factors shaping exposure, transmission, and microbial adaptation—may actually be the more informative for anticipating the emergence of novel human pathogens.

**Table 1:** Biological and eco-evolutionary factors that contribute to pathogen emergence.

Biological traits	Ecological and evolutionary conditions
Survival in the host	Host availability
Virulence factors	Exposure to pathogens
Host susceptibility	Genomic innovation

First, we must consider host availability and the rise of the human ‘host-scape’. Pathogen emergence depends not only on a microbe’s biological capacity to infect, but also on its opportunity to encounter and colonize suitable hosts. This opportunity is governed by host availability (the abundance, density, and spatial organization of potential hosts). For much of human evolutionary history, small, mobile groups of hunter-gatherers limited the opportunities for sustained pathogen transmission. This changed considerably when humans began to settle in agricultural communities roughly 10,000 years ago, a period that is sometimes referred to as the first epidemiological transition [3]. During this period, human populations became denser and so-called ‘crowd diseases’, such as tuberculosis and smallpox arose; other human diseases such as measles are believed to derive from animal pathogens and their emergence coincided with the rise of domestication [4].

The shift to sedentary life produced a dense and increasingly interconnected human host-scape. Populations grew, villages became towns and frequent contact among individuals facilitated the transmission of infectious agents that would previously have been unable to persist [5]. The resulting demographic transformation expanded the ecological niche available to pathogens, particularly those capable of human-to-human transmission. This increase in host availability created a broader foundation for microbial spillover and adaptation.

To quantify this transformation, consider that the majority of human pathogens probably evolved after this time. Using historical demographic data, I estimate the cumulative number of person-years from 10,000 BCE to 2025 CE to be approximately 1.62 × 10^12^. If one assumes that ~75% of known human bacterial pathogens emerged during this period, then more than 1,100 bacterial pathogens have emerged and persisted since the Neolithic period, giving a rate of bacterial pathogen acquisition of 6.99 × 10^−10^ per person-year, approximately one per 1.4 billion person-years.

Next to consider is the geographic distribution of microorganisms, which is also crucial in the acquisition of novel pathogens. In ecologically or socially isolated populations, the opportunities for contact with novel microbes are sharply limited. A spillover event in one region may never reach another if people and pathogens remain spatially separated. Historically, this isolation would have buffered many populations from epidemic diseases. In small or dispersed groups, even when a pathogen did cross the species barrier, it would often have failed to ignite widespread transmission due to limited host availability and movement. Without repeated introductions or long-distance movements, outbreaks of most emerging pathogens would have burned out quickly, unable to find immunologically naive hosts.

Globalization has changed this. In our interconnected world, characterized by extensive trade, tourism, migration and shipping, both people and pathogens move rapidly and frequently across geographic boundaries [6]. Today, more people may be exposed to a greater variety of pathogens than ever before, and pathogens are no longer bound by the ecological or social borders that once contained them. This substantially increases the probability that a spillover event will lead to sustained transmission. From the march of plague across medieval Eurasian trade routes, to the introduction of malaria to the Americas via the transatlantic slave trade, to the explosive spread of SARS-CoV-2 along commercial and travel corridors, pathogens have consistently followed the paths of human commerce.

Cholera illustrates this dynamic with unusual clarity. All seven recorded cholera pandemics have been linked to human movement. The current pandemic—the seventh—began in Java, Indonesia, in 1961 with the emergence of the *Vibrio cholerae* El Tor biotype as a globally spreading strain. Although circulating earlier in Sulawesi, its explosive spread began after being introduced by travelers from Makassar to a seaside community near Kendal, Java. Maritime links were key, including a regatta in Kuching that was attended by participants from Sulawesi, marking the start of the expansion of El Tor along coastal commercial routes [7,8]. It spread rapidly through Asia and into Africa and Europe in the 1960s and early 1970s. In 1991, it crossed the ocean (likely in contaminated ballast water discharged at a Peruvian port), triggering a regional epidemic that infected over 1 million people and killed approximately 10,000. The global trajectory of cholera underscores a broader point: in a connected world, trade and travel not only increase opportunities for exposure but also amplify the potential for sustained transmission.

Finally, we must consider that bacteria often acquire virulence and antimicrobial resistance through the activity of mobile genetic elements (MGEs). These elements (such as plasmids, transposons, and bacteriophages) enable genes to move laterally across strains, species, or even genera through horizontal gene transfer. In *V. cholerae*, key virulence determinants of the El Tor biotype, including the pathogenicity islands VSP-I and VSP-II, as well as the cholera toxin prophage, were acquired through the integration of MGEs [8]. Similarly, the opportunistic pathogens *Acinetobacter baumannii* and *Vibrio harveyi* have both acquired drug resistance through HGT [9,10]. As global trade, travel, and anthropogenic environmental changes (e.g., hydrological engineering or the homogenization of biodiversity through intentional or accidental species introduction) increasingly bring bacterial lineages into contact, they create unprecedented opportunities for genetic exchange between formerly isolated populations. It should be expected that this microbial mixing will accelerate the pace of pathogen innovation.

The ecological and evolutionary context of infectious disease emergence is changing at an unprecedented pace. While the biological prerequisites for pathogenicity—the capacity for infection, virulence factors, and host susceptibility—remain essential, these unfold within a landscape that is increasingly being shaped by global change. Given that the global human population is projected to reach 10.3 × 10^9^ by 2,100, and using an estimated rate of one enduring pathogen per 1.4 billion person-years, we might anticipate that roughly 504 additional bacterial pathogens may emerge by the end of the century. This may be a conservative estimate. Expanding global connectivity will increase the range and frequency of microbial exposures, while microbial mixing may accelerate genomic innovation through HGT, both of which increase the rate of emergence. At the same time, improvements in public health, biomedical infrastructure and changes in human–animal contact patterns have undoubtedly contributed to a declining per capita rate of pathogen acquisition. Although the traits identified by Casadevall [2] continue to define microbial pathogenicity, the dominant forces shaping emergence in the 21st century may now be ecological and evolutionary, external pressures that are being rapidly reshaped by human activity.

## Abbreviations

**:**
HGT: horizontal gene transfer
MGEs: mobile genetic elements
